# A neurostructural biomarker of dissociative amnesia: a hippocampal study in dissociative identity disorder

**DOI:** 10.1017/S0033291721002154

**Published:** 2023-02

**Authors:** Lora I. Dimitrova, Sophie L. Dean, Yolanda R. Schlumpf, Eline M. Vissia, Ellert R. S. Nijenhuis, Vasiliki Chatzi, Lutz Jäncke, Dick J. Veltman, Sima Chalavi, Antje A. T. S. Reinders

**Affiliations:** 1 Department of Psychological Medicine, Institute of Psychiatry, Psychology & Neuroscience, King's College London, London, UK; 2 Department of Psychiatry, Amsterdam UMC, Location VUmc, VU University Amsterdam, Amsterdam, The Netherlands; 3 Department of Psychosis Studies, Institute of Psychiatry, King's College London, London, UK; 4 Division of Neuropsychology, Department of Psychology, University of Zurich, Zurich, Switzerland; 5 Clienia Littenheid AG, Private Clinic for Psychiatry and Psychotherapy, Littenheid, Switzerland; 6 Heelzorg, Centre for Psychotrauma, Zwolle, The Netherlands; 7 Department of Biomedical Engineering, King's College London, London, UK; 8 Research Unit for Plasticity and Learning of the Healthy Aging Brain, University of Zurich, Zurich, Switzerland; 9 Movement Control and Neuroplasticity Research Group, Department of Movement Sciences, KU Leuven, Leuven, Belgium

**Keywords:** Dissociative experience scale, DES, childhood trauma, CA1, Freesurfer, dissociation

## Abstract

**Background:**

Little is known about the neural correlates of dissociative amnesia, a transdiagnostic symptom mostly present in the dissociative disorders and core characteristic of dissociative identity disorder (DID). Given the vital role of the hippocampus in memory, a prime candidate for investigation is whether total and/or subfield hippocampal volume can serve as biological markers of dissociative amnesia.

**Methods:**

A total of 75 women, 32 with DID and 43 matched healthy controls (HC), underwent structural magnetic resonance imaging (MRI). Using Freesurfer (version 6.0), volumes were extracted for bilateral global hippocampus, cornu ammonis (CA) 1–4, the granule cell molecular layer of the dentate gyrus (GC-ML-DG), fimbria, hippocampal−amygdaloid transition area (HATA), parasubiculum, presubiculum and subiculum. Analyses of covariance showed volumetric differences between DID and HC. Partial correlations exhibited relationships between the three factors of the dissociative experience scale scores (dissociative amnesia, absorption, depersonalisation/derealisation) and traumatisation measures with hippocampal global and subfield volumes.

**Results:**

Hippocampal volumes were found to be smaller in DID as compared with HC in bilateral global hippocampus and bilateral CA1, right CA4, right GC-ML-DG, and left presubiculum. Dissociative amnesia was the only dissociative symptom that correlated uniquely and significantly with reduced bilateral hippocampal CA1 subfield volumes. Regarding traumatisation, only emotional neglect correlated negatively with bilateral global hippocampus, bilateral CA1, CA4 and GC-ML-DG, and right CA3.

**Conclusion:**

We propose decreased CA1 volume as a biomarker for dissociative amnesia. We also propose that traumatisation, specifically emotional neglect, is interlinked with dissociative amnesia in having a detrimental effect on hippocampal volume.

## Introduction

Dissociative amnesia is a dissociative symptom common in the dissociative disorders and is characterised by recurrent gaps in recalling everyday events and/or of important personal (trauma-related) information, distinct from ordinary forgetting. The most severe of the dissociative disorders is dissociative identity disorder (DID). DID is a debilitating psychiatric condition and is related to, among others, alternating states of consciousness and distinct personality states with changing access to autobiographical information (American Psychiatric Association, 2013). Although dissociative amnesia is a core symptom of DID and of other dissociative disorders little is known about its neurobiological foundations.

A few studies have found a negative correlation between dissociative symptoms and hippocampal volume. Ehling, Nijenhuis, and Krikke (2008) found bilateral global hippocampal volume reductions in individuals with DID, which negatively correlated with dissociative symptoms. Similarly, Chalavi et al. (2015b) found evidence for hippocampal global and subfield volume reductions in relation to dissociative symptoms and/or traumatisation. Left hippocampal volume reduction has further been associated with dissociative symptoms in individuals who suffered childhood sexual abuse as compared with healthy controls (HC) (Stein, Koverola, Hanna, Torchia, & McClarty, 1997) indicating that early traumatisation is a potential mediator of dissociative symptoms. Adverse childhood experiences further increase in the likelihood of childhood autobiographical memory deficits (Brown et al., 2007). Amnesia has also been linked to the presence of psychological stress and/or traumatisation (Markowitsch & Staniloiu, 2012; Staniloiu & Markowitsch, 2012). On the other hand, there is also information to suggest absence of hippocampal volume reductions in individuals with a dissociative disorder (Weniger, Lange, Sachsse, & Irle, 2008) and a study by Mutluer et al. (2018) did not show significant correlations between hippocampal volume and dissociative symptoms in a group of PTSD patients. Thus, although most studies reported decreased hippocampal volume, evidence for a negative correlation between hippocampal volume and dissociative symptoms is not fully consistent. Variations of findings across these studies may be driven by the low numbers of participants and/or inconsistent measurement of the dissociative symptoms. Therefore, there is a need for further research into the role of the hippocampus in dissociative amnesia in DID.

The hippocampus plays an integral role in consolidating long-term memories and in learning (Preston & Eichenbaum, 2014). As such, it is relevant to consider the hippocampus in relation to dissociative amnesia. The hippocampus consists of different anatomical subfields, namely the cornu ammonis 1, 2, 3 and 4 (CA1–4), the parasubiculum, presubiculum, subiculum and the hippocampal tail, as well as other regions, namely the granule cell molecular layer of the dentate gyrus (GC-ML-DG), the hippocampal–amygdaloid transition area (HATA), the fimbria, the hippocampal fissure and the molecular layer of the hippocampus (Amaral & Lavenex, 2006). Research has shown that excessive stress from traumatic experiences, particularly childhood traumatic experiences, contributes to the dysregulation of the hypothalamic pituitary adrenal axis (Kuhlman, Vargas, Geiss, & Lopez-Duran, 2017; Shea, Walsh, MacMillan, & Steiner, 2004), which may damage hippocampal function and structure (Bidzan, 2017; Teicher, Anderson, & Polcari, 2012; Woon, Sood, & Hedges, 2010). Certain hippocampal regions have been identified as more important in memory and amnesia including the CA1 (Bartsch, Döhring, Rohr, Jansen, & Deuschl, 2011; Spiegel et al., 2017). CA1 is a subfield known to be critically involved in the process of memory consolidation and could be involved in dissociative amnesia (Spiegel et al., 2017). Vulnerability and susceptibility of the CA1 to the detrimental effects of stress could therefore lead to dissociative amnesia. To date, only one study has investigated the global volume of the hippocampus and of its composite smaller regions in relation to dissociative symptoms in DID participants and showed a negative correlation between total dissociation scores and hippocampal regions (Chalavi et al., 2015b).

Dissociative symptomatology is of a complex nature. Many different theoretical and definitional frameworks have endeavoured to conceptualise it (Dell & O'Neil, 2009; Dorahy et al., 2014; Holmes et al., 2005; Nijenhuis, 2015; Reinders & Veltman, 2020; Şar, Dorahy, & Krüger, 2017). Most research into pathological dissociation has used total scores from the dissociative experience scale (DES) (Bernstein & Putnam, 1986) to assess psychoform dissociative symptoms in relation to, for example, cognitive functioning or neurobiology (Roydeva & Reinders, 2021). However, the DES is considered to have a three-factor structure, the factors being dissociative amnesia, absorption and depersonalisation/derealisation. Increasingly evidence shows that amnesia, absorption and depersonalisation/derealisation are separate unique dissociative components that need to be examined as separate entities in research (Lyssenko et al., 2018; Soffer-Dudek, Lassri, Soffer-Dudek, & Shahar, 2015). For instance, unlike amnesia and depersonalisation/derealisation, absorption has been found to be normally distributed among healthy populations (Soffer-Dudek et al., 2015) and it is suggested that absorption involves an alternation of consciousness that is not dissociative (Nijenhuis, Van der Hart, & Steele, 2002b). Therefore, this raises concerns as to whether absorption could diminish statistical effects when pathological dissociation is examined as a cumulative overall phenomenon. Consequently, it is important to assess individually the core diagnostic features of DID and their neural correlates as well as to investigate the three dissociative constructs in relation to hippocampal morphology, which could provide a comprehensive understanding of the specificity of structural alterations.

The aims of the current study are to extend previous research by Chalavi et al. (2015b) by increasing the sample size and to investigate the distinct contributions of the three separate DES factors, that is amnesia, absorption and depersonalisation/derealisation, to decreases in hippocampal global and subfield volumes in a large sample of individuals with DID. In addition, due to the inter-linked nature of trauma and dissociation, we aim to investigate the global volume of the hippocampus and of its composite smaller regions in relation to self-report measures of traumatisation related to emotional abuse, emotional neglect, physical abuse, sexual abuse and sexual harassment. We hypothesise that hippocampal global and subfield volumes will be smaller in individuals with DID as compared to HC and that within the DID group the global and subfield hippocampal volumes will negatively correlate with higher severity of dissociative amnesia as well as with greater traumatisation.

## Methods

### Design and participants

Data of a total of 75 women (only female participants with DID volunteered) were included in the current study which follows a between-group research design: 32 female volunteers with DID and 43 HC matched for age, gender, years of education and ethnicity. Data were collected in the Netherlands in the University Medical Centre in Groningen (UMCG) and the Amsterdam Medical Centre (AMC), and in Switzerland at the University Hospital in Zurich. Participant information has been detailed previously (Chalavi et al., 2015a, 2015b; Reinders et al., 2018, 2019; Schlumpf et al., 2013, 2014). The DID participants were recruited from psychiatric departments, outpatient psychotherapists and psychiatrists. Initial diagnosis fulfilled DSM-IV criteria and was confirmed by trained clinicians with the Structural Clinical Interview for DSM-IV Dissociative Disorders (SCID-D) (Steinberg, 1993). Twenty-nine volunteers with DID had a co-morbid diagnosis of PTSD and three had PTSD in remission. Additional co-morbidity was confirmed by participants and their personal therapists based on DSM-IV classification (American Psychiatric Association, 1994), see for details Reinders et al. (2018, 2019) and online Supplementary Table S1. The HC group was recruited through local newspaper advertisements. Exclusion criteria for all participants included age outside the range of 18–65, pregnancy, systemic or neurological illness, claustrophobia, metal implants in the body and substance abuse. Additional exclusion criteria for the HC group included the presence of dissociative symptoms and a history of trauma, past or current psychiatric disorders and medication use (see for details Chalavi et al. 2015a, 2015b; Reinders et al. 2018; Schlumpf et al. 2013, 2014). HC were required to score below 25 on the DES. The DES is a 28-item self-report screening tool for dissociative disorders which is often used in clinical research and as a basis for guiding diagnosis in the clinical interview (Carlson & Putnam, 1993). The DES scale has received meta-analytic validation (Van Ijzendoorn & Schuengel, 1996). The HC were also required to have a score below and 29 on the Somatoform Dissociation Questionnaire (Nijenhuis, Spinhoven, Van Dyck, Van der Hart, & Vanderlinden, 1996). Traumatic experiences were measured with the traumatic experience checklist (TEC), a self-report measure of potentially traumatising events (Nijenhuis, Van der Hart, & Kruger, 2002a). TEC total scores were previously calculated for each category of abuse, namely emotional neglect, emotional abuse, physical abuse, sexual abuse and sexual harassment (Reinders et al., 2018). As expected and previously reported (Reinders et al., 2018, 2019) dissociative symptoms and traumatic experiences were significantly higher in the DID group (all data obtained in the predominant personality state) than in the HC group (all *p*-values <0.001, see for details online Supplementary Table S1).

### Ethical considerations

All participants gave informed written consent in accordance with the Declaration of Helsinki and as dictated by ethical requirements of the Medical Ethical Committees of UMCG (Reference number: METC2008.211) and the AMC (Reference number: MEC09/155), and by the cantonal ethical commission of Zurich (Kantonale Ethikkommission Zürich; reference number: E-13/2008). All participants were given the right to withdraw and were fully debriefed in line with the ethical requirements of the Declaration of Helsinki (World Medical Association, 2013).

### Magnetic resonance imaging acquisition

Magnetic resonance imaging (MRI) data were collected using 3-T Philips whole-body scanners (Philips Medical Systems, Best, Netherlands) from centres in the Netherlands (The AMC, and the UMCG) and Switzerland (University Hospital in Zurich, UHZ). Ratios of DID to HC participants were approximately equal across the centres (10 DID participants and 17 HC included from the UMCG, 7 DID participants and 11 HC from the AMC, and 15 DID participants and 15 HC from Zurich). The number within each group did not differ across centres (χ^2^ = 1.01, *p* = 0.603). An optimised T1-weighted anatomical MRI protocol with the following settings was utilised which has been demonstrated to have high reproducibility within and between centres (Chalavi, Simmons, Dijkstra, Barker, & Reinders, 2012; 3D MPRAGE, repetition time = 9.95 ms, echo time = 5.6 ms, flip-angle = 8°, voxel size = 1 × 1 × 1 mm^3^, number of slices = 160, total scan time = 10 m 14 s).

### Volumetric analysis

**Fig. 1.**
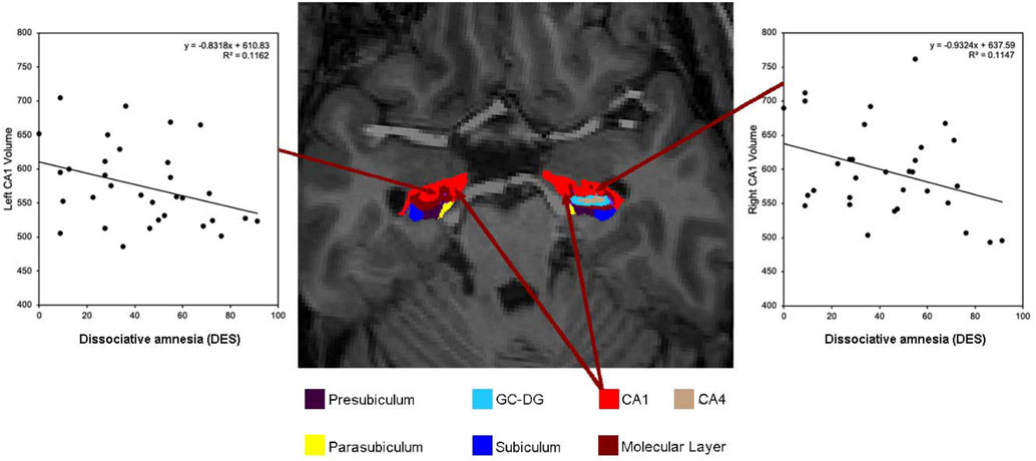
Axial slice of the hippocampus from a DID participant with scatter plots showing the relationship between increased dissociative amnesia scores as assessed by the dissociative experiences scale (DES) and decreased bilateral CA1 volume.

MRI data were processed using FreeSurfer version 6.0. Following full surface reconstruction and volumetric segmentation (Dale, Fischl, & Sereno, 1999; Dale & Sereno, 1993; Fischl & Dale, 2000; Fischl, Liu, & Dale, 2001; Fischl, Sereno, & Dale, 1999a; Fischl, Sereno, Tootell, & Dale, 1999b; Fischl et al., 2002, 2004a, 2004b; Han et al., 2006; Jovicich et al., 2006; Segonne et al., 2004; Yeo et al., 2010a, 2010b – for full details, see Fischl, 2012; Fischl et al., 2002), volumetric measures for the global hippocampus, CA1, CA3, CA4, fimbria, HATA, subiculum, GC-DG, parasubiculum and presubiculum, for each hemisphere (Fig. 1), in addition to total intercranial volume (TIV), were extracted. For one participant from the HC group FreeSurfer was not able to complete the hippocampal segmentation, and therefore this participant was excluded from subsequent statistical analyses.

### Dissociative amnesia

Dissociative amnesia was measured as part of the DES. In addition to total dissociation the three core dissociative symptoms, namely absorption, amnesia and depersonalisation/derealisation were calculated.

### Statistical analysis

All analyses were performed using SPSS (v25) (IBM Corp., 2017). To confirm the findings of Chalavi et al. (2015b) ‘that hippocampal global and subfield volumes are smaller in individuals with DID compared to a control group’, in the extended sample we performed a similar group comparison between DID and HC participants. If group differences are found between the two groups further correlation analyses are warranted. Then independent samples *t* tests were used to test differences between the groups in age, education and total dissociation, absorption, amnesia and depersonalisation/derealisation. Mann–Whitney U tests were applied to analyse group differences in traumatisation measures because of skewness in the data. Analyses were then conducted within the DID group only to assess dissociation and traumatisation measures and maintain the validity of the results to pathological samples.

### Hippocampal volumes

Between-group differences in hippocampal volumes for each hemisphere were examined with analyses of covariance (ANCOVA). Hippocampal volumes acted as the dependant variable, group and centre as fixed categorical effects, and age and estimated TIV as continuous covariates. Controlling for TIV allows the examination of volumetric changes with respect to maximal adult brain size (O'Brien et al., 2012). Significant group differences were assessed by comparing the estimated marginal means of the main effects in post-hoc with the Bonferroni confidence interval adjustment. Partial eta squared measures of effect size were reported for main effects, interpreted as small (0.01), medium (0.06) or large (0.14) (Cohen, 1988). Cohen's *d* was calculated for the post-hoc pairwise comparisons by dividing the adjusted mean difference by the square root of the mean squared (MS) error from analysis of variance (Howell, 2010), and were interpreted using Cohen (1988) benchmark of small (0.2), medium (0.5) and large (0.8).

### Hippocampal volumes and dissociation and traumatisation

Hippocampal volume in relation to dissociation and traumatisation was evaluated within the DID group only to preserve validity. Including the HC group would lead to spurious results because per exclusion criteria the HC are free from dissociation symptoms (see for discussion Chalavi et al. 2015a, 2015b; Dimitrova et al. 2020; Preacher, Rucker, MacCallum, & Nicewander, 2005). Partial correlations were conducted between volumetric measures and dissociation total and subscale measures, controlling for age and TIV. The Kolmogorov–Smirnov test detected no deviations from normality in the dissociation measures within the DID group. Within the DID group, the associations of total TEC scores and subscale scores of abuse categories with hippocampal volumes for each hemisphere were explored with partial correlations, controlling for age and TIV. Due to the ordinal nature of the TEC data and lack of normality as assessed by the Kolmogorov–Smirnov test (all *p* < 0.05), Spearman's partial correlations were conducted using a script in the SPSS syntax editor.

Results were controlled for co-morbidity by repeating the partial correlations and adding the co-morbid diagnosis as a third variable to the analyses, in a similar manner as Chalavi et al. (2015a). This meant that the correlations between dissociation measures and hippocampal volumes were controlled for age, TIV and co-morbidity. Additionally, we created a ‘total’ covariate variable, data coded with 1 if any co-morbidity was present, and a 0 if none was present. PTSD was not included as a co-morbidity because all individuals with DID also had a diagnosis of PTSD (in remission). Adding PTSD as a covariate would therefore invalidate the analyses.

## Results

### Hippocampal volumes

Bilateral hippocampal global volume was significantly smaller in the DID group compared with the HC group (left: *F*(1,69) = 6.183, *p* = 0.015, *η_p_*^2^ = 0.087, *d* = 0.61; right: *F*(1,66) = 5.425, *p* = 0.023, *η_p_*^2^ = 0.076, *d* = 0.57). Regarding hippocampal subfield and region volumes, smaller volumes for the DID group compared with the HC group were found for bilateral CA1 (left: *F*(1,66) = 4.785, *p* = 0.032, *η_p_*^2^ = 0.068, *d* = 0.53; right: *F*(1,66) = 5.812, *p* = 0.019, *η_p_*^2^ = 0.081, *d* = 0.59), right CA4 (*F*(1,65) = 4.187, *p* = 0.045, *η_p_*^2^ = 0.061, *d* = 0.50), right GC-ML-DG (*F*(1,65) = 4.130, *p* = 0.046, *η_p_*^2^ = 0.060, *d* = 0.50) and left presubiculum (*F*(1,65) = 5.663, *p* = 0.020 *η_p_*^2^ = 0.080, *d* = 0.58). Details are provided in online Supplementary Table S2.

### Hippocampal volumes and dissociation

**Fig. 2.**
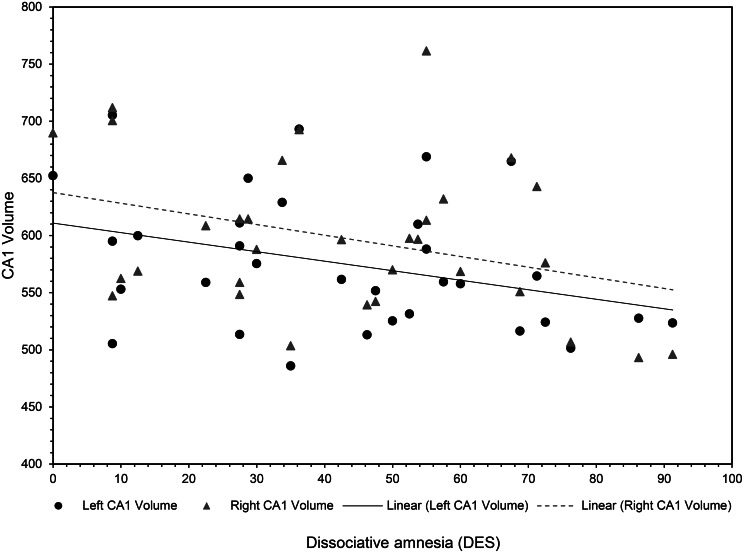
Scatter plot showing the relationship between dissociative amnesia as assessed by the DES and bilateral CA1 volume. The correlations indicate a reduction in volume for both hemispheres with higher severity of dissociative amnesia.

Dissociative amnesia, absorption, depersonalisation/derealisation scores as well as total DES scores correlated with hippocampal global and subfield volumes. However, only dissociative amnesia and total DES scores correlated significantly, that is, between dissociative amnesia and reduced bilateral hippocampal CA1 subfield volumes (left: *r* = −0.396, *p* = 0.030; right: *r* = −0.363, *p* = 0.049) and between total DES scores and left CA1 subfield (*r* = −0.369, *p* = 0.045). Results are illustrated in Figs 1, 2 and online Supplementary Fig. S1. There were no significant correlations between symptoms of absorption or depersonalisation/derealisation scores and hippocampal volumes. Details are provided in online Supplementary Table S3. Online Supplementary Table S4 contains the results corrected for co-morbidity using covariate analyses.

### Hippocampal volumes and traumatisation

Measures of traumatic experiences correlated with hippocampal volumes. Negative correlations were found between emotional neglect and bilateral global hippocampus (left: *r* = −0.442, *p* = 0.021; right: *r* = −0.431, *p* = 0.025), bilateral CA1 (left: *r* = −0.408, *p* = 0.035; right: *r* = −0.392, *p* = 0.043), right CA3 (*r* = −0.411, *p* = 0.033), bilateral CA4 (left: *r* = −0.446, *p* = 0.020; right: *r* = −0.462, *p* = 0.017) and bilateral GC-ML-DG (left: *r* = −0.460, *p* = 0.016; right: *r* = −0.469, *p* = 0.016). There were no significant negative correlations between hippocampal volumes and emotional, physical and sexual abuse subscales, sexual harassment and total traumatisation scores. Details are provided in online Supplementary Table S5.

## Discussion

The current study is the first to investigate decreased hippocampal global and subfield volumes in relation to dissociative amnesia, absorption and depersonalisation/derealisation symptoms in the largest cohort of individuals with DID to date. Our most important finding is that in the DID group only dissociative amnesia and total dissociation symptom scores and not absorption or depersonalisation/derealisation correlated significantly and negatively with hippocampal volume. Our second most important finding is that these negative correlations were only found for the CA1 hippocampal subfield.

We propose that the volume of CA1 can serve as a biomarker for dissociative amnesia because only dissociative amnesia and not absorption or depersonalisation/derealisation correlated with smaller CA1 hippocampal subfield volume in individuals with DID. Research has previously shown that the total DES score correlates negatively with hippocampal volume in patients with DID and PTSD (Chalavi et al., 2015b; Ehling et al., 2008; Mutluer et al., 2018; Stein et al., 1997), but our results indicate that this effect might be driven by dissociative amnesia. Further to this, the association of CA1 with dissociative amnesia remained significant even after controlling for co-morbidity. This indicates that CA1 volume reduction is primarily driven by dissociative amnesia in DID, not other disorders, and presents a specific dissociative disorder effect.

Our novel findings can be linked to amnesia and memory because damage to hippocampal subfield CA1 has been shown to lead to memory impairments (Bartsch et al., 2015, 2011; Ocampo, Squire, & Clark, 2017). Furthermore, CA1 has been found to be of particular importance for autobiographical memories (Bartsch et al., 2011), which constitute building blocks of a person's identity. Moreover, CA1 impairments have been linked to damage in extinction learning (the gradual decrease in response to a conditioned stimulus) in individuals presenting trauma, implicating accurate context evaluation of non-threatening settings (Chen et al., 2018). Consequently, damage to or lack of maturation of CA1 regions may be related to memory disturbances. The CA1 projects to the medial prefrontal cortex and the orbitofrontal frontal cortex (Zhong, Yukie, & Rockland, 2006) and it could be speculated that damage to the CA1 may contribute to dissociation mechanisms and the formation of dissociative personality states (Forrest, 2001).

We also found a link between the severity of childhood traumatisation, specifically emotional neglect and reductions in hippocampal volume including CA1. In support of our finding research has demonstrated an association between emotional neglect and dissociation severity accentuating the relationship between traumatisation, dissociation and hippocampal volume reductions (Şar, 2011; Şar, Akyüz, & Doğan, 2007; Schalinski et al., 2016; Schimmenti, 2016). CA1 impairment in relation to childhood traumatisation can possibly lead to fragmentation of the mind and a scattered sense of self (Brown et al., 2007). Interestingly, Huntjens, Dorahy, and van Wees-Cieraad (2013) present lack of self-referential processing as a possible mechanism to explain the link between dissociation and fragmentation of the mind. Furthermore, research has shown memory source misattribution as a specific cognitive characteristic of dissociation, particularly for dissociative amnesia (Chiu et al., 2016, 2019). The association between dissociative symptoms and misattributing self-generated representations as an external doing suggests an amnestic barrier regarding access to self-relevant information. Chiu et al. (2016) state that this misattribution error could not be fully explained by intellectual function and general psychopathology, which suggests that this cognitive blockage of information is a specific cognitive characteristic of dissociation. They also found that source misattribution of self-generated representations correlated significantly with DES assessed dissociative amnesia (Chiu et al., 2016). Furthermore, it has been found that there is a link between dissociation proneness and reduced self-reference ability, particularly for individuals with high dissociation proneness and high childhood relational trauma (Chiu et al., 2019). The inability to establish a self-referential perspective during traumatisation prevents processing the experience as own and self-relevant and inhibits assimilating those memories into the autobiographical memory base (Huntjens et al., 2013). This supports the altered sense of individuality prevalent in DID and the amnestic blockage of information as evident with dissociative amnesia. Furthermore, there are consistent reports on associations between abuse and dissociation severity across a range of clinical presentations including PTSD, borderline personality disorder (BPD) and psychosis (Schalinski & Teicher, 2015; Schalinski et al., 2016). A multitude of factors interplay with dissociation severity including the relationship to the perpetrator (Plattner et al., 2003), attachment style (Kong, Kang, Oh, & Kim, 2018) and genetics (Dackis, Rogosch, Oshri, & Cicchetti, 2012; Savitz et al., 2008; Wolf et al., 2014).

Our study is, to the best of our knowledge, the first to show that decreased hippocampal subfield volume is related to dissociative amnesia and is interlinked with emotional neglect. These findings need to be confirmed in future research because emotional neglect is the only nonviolent traumatisation measure, while past research has emphasised the importance of physical and sexual abuse in dissociative disorders (Mutluer et al., 2018; Stein et al., 1997; Twaite & Rodriguez-Srednicki, 2004). For instance, there are reports correlating dissociative symptoms with various adverse events, including emotional neglect by family of origin, in addition to research suggesting emotional neglect (e.g. parental unavailability) during childhood as a major predictor for developing a dissociative disorder (Dutra, Bureau, Holmes, Lyubchik, & Lyons-Ruth, 2009; Lyons-Ruth, Dutra, Schuder, & Bianchi, 2006; Nijenhuis, Spinhoven, Van Dyck, Van der Hart, & Vanderlinden, 1998; Ogawa, Sroufe, Weinfield, Carlson, & Egeland, 1997).

Our finding of the importance of CA1 in dissociative amnesia is of interest as it provides insight into further understanding biomarkers of dissociative disorders. Furthermore, by showing the variation of significance of the three subscales of the DES, that is amnesia, absorption and depersonalisation/derealisation, we emphasise with others (Lyssenko et al., 2018; Soffer-Dudek et al., 2015) the importance of examining the subscales as separate entities and not as a total cumulative unit. By identifying a region of interest that is linked to dissociative amnesia we provide evidence of implications in the clinical realm (Reinders & Veltman, 2020). Our study has possible clinical relevance because there is some evidence to suggest hippocampal volume increase/recovery after medication (Vermetten, Vythilingam, Southwick, Charney, & Bremner, 2003) and with phase-oriented psychotherapy (Ehling et al., 2008), but not with brief eclectic psychotherapy (Lindauer et al., 2005). However, to date, there is little evidence regarding treatment outcomes for DID and future research could investigate the role of CA1 hippocampal subfield volume during treatment of and recovery from dissociative disorders. We also recommend that future studies provide more precise results in terms of hippocampal head, body and tail and their respective connectivity in a larger sample of DID participants. This is of interest because the anterior hippocampus has been associated with trauma-related memories and intrinsic functional connectivity with the amygdala, nucleus accumbens, medial prefrontal cortex, posterior cingulate cortex, midline thalamus and periventricular hypothalamus (Abdallah et al., 2017; Blessing, Beissner, Schumann, Brünner, & Bär, 2016). Future studies could also formulate hypotheses about and include a wider range of measures of dissociative symptomatology such as positive and negative dissociative symptoms (Spiegel et al., 2013), detachment and compartmentalisation (Cardeña & Carlson, 2011) and trait and state dissociation (Roydeva & Reinders, 2021).

Our study presents the following limitations. Firstly, our study only included participants with DID and future research should assess dissociative amnesia across other psychiatric disorders to confirm our proposal of CA1 as a biomarker for dissociative amnesia. Furthermore, smaller hippocampal volume has been found in various mental disorders with and without dissociative symptoms (Belli, 2014; Chalavi et al., 2015b; Luoni, Agosti, Crugnola, Rossi, & Termine, 2018; Roydeva & Reinders, 2021; van Huijstee & Vermetten, 2017). Due to the polysymptomatic presentation of symptoms in DID it is important that future research controls for comorbidity findings. Secondly, our study is limited by the exclusively female sample. Therefore our results may not extend to men with DID. Nevertheless, by focusing on women exclusively, our study design is optimised to minimise the neuroanatomical and clinical heterogeneity that could have been introduced by analysing data across gender categories (Reinders et al., 2018). It can be argued that seeing a correlation of hippocampal volume and (self-report) severity of amnesia in the DID group is evidence that this neural substrate is relevant to the severity of amnesia in DID, rather than the presence of dissociative amnesia because amnesia is a defining symptom of DID and is therefore expected to be present in all participating individuals with DID. Although this is indeed the case and symptom scores of absorption and depersonalisation/derealisation are 4–5% higher than for dissociative amnesia, our proposal of decreased CA1 volume as a biomarker for dissociative amnesia is also based on the *absence* of a correlation between hippocampal (sub-)volumes and symptoms of absorption and depersonalisation/derealisation instead of only the *presence* of dissociative amnesia. Therefore, we propose that our findings are of interest for neurobiological biomarker research.

In conclusion, our study proposes decreased CA1 volume as a biomarker for dissociative amnesia. We also propose that emotional neglect is interlinked with dissociative amnesia in having a detrimental effect on hippocampal subfield volume.

## Supplementary material

For supplementary material accompanying this paper visit https://doi.org/10.1017/S0033291721002154. click here to view supplementary material
