# Supplementary material for: A neurostructural biomarker of dissociative amnesia: a hippocampal study in dissociative identity disorder
Source: Psychol Med. 2021 Jun 24;53(3):805–13. doi: 10.1017/S0033291721002154 (PMC9975991; doi:10.1017/S0033291721002154)
Supplement: Supplementary file 1 [file S0033291721002154sup001.docx]

**SUPPLEMENTARY MATERIALS**

| **Table S1.** *Demographic characteristics and clinical comparisons between DID and HC.* | | | | |
| --- | --- | --- | --- | --- |
| **Demographics** | **Mean (SD)** | | **t-test (df) / *U*** | **Sig.** |
|  | **DID (n = 32)** | **HC (n = 42)** |  |  |
| **Age** | 43.56 (9.34) | 42.17 (11.69) | -0.55 (72) | .581 |
| **Education** | 14.31 (2.04) | 14.90 (1.59) | 1.36 (57.11)^c^ | .180 |
| **Medication**^a^ |  |  |  |  |
| *anti-psychotics: n (typical, atypical)* | (4, 12) | 0 | NA |  |
| *anti-epileptics: n* | 5 | 0 | NA |  |
| *anti-depressants: n* | 21 | 0 | NA |  |
| **Comorbidity** *PTSD: n (current, in remission)* | (29, 3) | 0 | NA |  |
| ***Comorbidity other*** ^b^ |  |  |  |  |
| *somatoform disorder: n* | 2 | 0 | NA |  |
| *depression: n (chronic, recurrent)* | (1, 10) | 0 | NA |  |
| *dysthymic disorder: n* | 1 | 0 | NA |  |
| *specific phobias: n* | 3 | 0 | NA |  |
| *panic disorder: n* | 3 | 0 | NA |  |
| *anxiety disorder: n* | 1 | 0 | NA |  |
| *obsessive-compulsive disorder: n* | 1 | 0 | NA |  |
| *personality disorders: n (NOS, mixed, BPD, dependent, histrionic)* | (2,2,5,1,1) | 0 | NA |  |
| *eating disorder: n* | 3 | 0 | NA |  |
| *sleeping disorder: n* | 2 | 0 | NA |  |
| *catalepsy: n* | 1 | 0 | NA |  |
| *psychogenic seizures: n* | 1 | 0 | NA |  |
| *attention deficit disorder: n* | 1 | 0 | NA |  |
| *no other comorbid disorder: n* | 13 | 0 | NA |  |
| **Dissociative symptoms** |  |  |  |  |
| ***Dissociation Experience Scale (DES)*** |  |  |  |  |
| *amnesia* | 42.85 (24.37) | 2.83 (4.30) | -9.18 (32.47)^c^ | <.001 |
| *absorption* | 50.24 (18.30) | 9.21 (7.46) | -11.96 (38.88)^c^ | <.001 |
| *depersonalisation/derealisation* | 52.03 (20.12) | 1.83 (3.05) | -13.99 (32.09)^c^ | <.001 |
| *total* | 50.14 (17.94) | 6.26 (5.12)^d^ | -13.42 (34.96)^c^ | <.001 |
| **Traumatic Experiences Checklist (TEC)** |  |  |  |  |
| *emotional neglect* | 12.28 (2.58)^b^ | 2.60 (4.39) | 1110.50^e^ | <.001 |
| *emotional abuse* | 11.86 (3.26)^b^ | 1.53 (3.23) | 1164.00^e^ | <.001 |
| *physical abuse* | 11.69 (3.63)^b^ | 0.60 (1.99) | 1141.00^e^ | <.001 |
| *sexual harassment* | 10.07 (4.29)^b^ | 0.52 (1.57) | 1170.00^e^ | <.001 |
| *sexual abuse* | 10.38 (4.41)^f^ | 0.07 (0.34) | 1174.00^e^ | <.001 |
| *total* | 18.45 (3.97)^b^ | 3.14 (2.78) | 1217.00^e^ | <.001 |
| *Note*: DID = Dissociative identity disorder; HC = Healthy controls; PTSD = post-traumatic stress disorder; NOS = not otherwise specified; BPD = borderline personality disorder; df = degrees of freedom; U = Mann-Whitney U test result; Sig. = significant p-value; NA = not applicable  ^a^ = past and present medication use  ^b^ = DID n = 29 due to missing values  ^c^ = equal variances not assumed  ^d^ = HC n = 41 due to missing values  ^e^ = Mann-Whitney U used due to skewness in TEC scores  ^f^ = DID n = 28 due to missing values | | | | |

| **Table S2.** *Descriptive statistics and ANCOVA analyses between DID and HC on hippocampal volume.* | | | | | | | | | |
| --- | --- | --- | --- | --- | --- | --- | --- | --- | --- |
|  | **Descriptive statistics** | |  | **Comparisons** | | | | | |
|  | **Mean (SD)** | |  | **ANCOVA** | | | | | |
|  | **DID (n=32)** | **HC (n=42)** |  | ***F*(df)** | **Sig.** | **η_p_^2^** | **Mean difference (mm^3^)** | **CI** | ***d*** |
| **Hippocampal volumes** | | | | | | | | | |
| *Left hippocampus* | 3180.70 (291.15) | 3318.83 (236.30) |  | 6.183 (1,69) | **.015** | .087 | 152.25 | 29.963, 274.528 | .61 |
| *Right hippocampus* | 3249.05 (287.54) | 3368.55 (230.92) |  | 5.425 (1,66) | **.023** | .076 | 142.55 | 20.353, 264.737 | .57 |
| *Left CA1* | 575.19 (59.45) | 603.85 (55.45) |  | 4.785 (1,66) | **.032** | .068 | 30.55 | 2.667, 58.431 | .53 |
| *Right CA1* | 597.63 (67.08) | 628.95 (55.64) |  | 5.812 (1,66) | **.019** | .081 | 35.68 | 6.130, 65.228 | .59 |
| *Left CA3* | 187.37 (24.50) | 194.39 (23.52) |  | 1.745 (1,66) | n.s. | - | - | - | - |
| *Right CA3* | 202.29 (24.78) | 211.56 (27.32) |  | 3.702 (1,66) | .059^ | .053 | 12.00 | -0.452, 24.443 | .47 |
| *Left CA4* | 239.19 (26.89) | 248.57 (23.65) |  | 3.467 (1,66) | .067^ | .050 | 10.81 | -0.782, 22.411 | .45 |
| *Right CA4* | 240.82 (24.22) | 250.47 (22.87) |  | 4.187 (1,65) | **.045** | .061 | 11.44 | 0.274, 22.608 | .50 |
| *Left GC-ML-DG* | 278.52 (31.49) | 288.89 (28.07) |  | 2.962 (1,66) | .090^ | .043 | 11.83 | -1.894, 25.561 | .42 |
| *Right GC-ML-DG* | 280.24 (27.79) | 291.62 (26.44) |  | 4.130 (1,65) | **.046** | .060 | 13.23 | 0.229, 26.224 | .50 |
| *Left subiculum* | 400.39 (46.34) | 417.13 (39.87) |  | 2.883 (1,66) | .094^ | .042 | 17.49 | -3.077, 38.047 | .41 |
| *Right subiculum* | 402.17 (37.81) | 417.02 (30.85) |  | 3.628 (1,66) | .061^ | .052 | 15.73 | -0.758, 32.214 | .46 |
| *Left presubiculum* | 290.15 (30.63) | 305.59 (26.03) |  | 5.663 (1,65) | **.020** | .080 | 15.58 | 2.505, 28.655 | .58 |
| *Right presubiculum* | 284.55 (27.20) | 294.85 (32.72) |  | 1.558 (1,66) | n.s. | - | - | - | - |
| *Left parasubiculum* | 56.83 (10.12) | 58.61 (8.32) |  | 0.590 (1,66) | n.s. | - | - | - | - |
| *Right parasubiculum* | 55.54 (8.71) | 55.79 (7.60) |  | 0.003 (1,66) | n.s. | - | - | - | - |
| *Left HATA* | 56.80 (7.08) | 59.38 (7.93) |  | 1.973 (1,66) | n.s. | - | - | - | - |
| *Right HATA* | 58.85 (7.63) | 62.49 (8.83) |  | 2.192 (1,66) | n.s. | - | - | - | - |
| *Left fimbria* | 78.28 (13.34) | 79.56 (16.50) |  | 0 (1.66) | n.s. | - | - | - | - |
| *Right fimbria* | 73.66 (17.05) | 73.92 (17.29) |  | 0.535 (1,66) | n.s. | - | - | - | - |
| *Note*: DID = Dissociative Identity Disorder; HC = Healthy Controls; CI = Confidence intervals; CA = cornu ammonis; GC-ML-DG = granule cell molecular layer of the dentate gyrus; HATA = hippocampal-amygdaloid transition area; n.s. = not significant; η_p_^2^ = partial Eta squared**;** Sig. = significant p-value; d = Cohen’s d; ^ = 0.05<*p*≤0.1 | | | | | | | | | |

| **Table S3**. *Correlations of hippocampal volumes with severity of dissociative symptoms within DID.* | | | | | | | | |
| --- | --- | --- | --- | --- | --- | --- | --- | --- |
|  | **Amnesia** | | **Absorption** | | **DP/DR** | | **DES total** | |
|  | ***r*** | **Sig.** | ***r*** | **Sig.** | ***r*** | **Sig.** | ***r*** | **Sig.** |
| **Hippocampal volumes** | | | | | | | | |
| *Left hippocampus* | -.193 | .316 | -.046 | .812 | .027 | .889 | -.111 | .565 |
| *Right hippocampus* | -.339 | .067^ | -.181 | .338 | -.098 | .606 | -.260 | .166 |
| *Left CA1* | **-.396** | **.030** | -.264 | .159 | -.191 | .311 | **-.369** | **.045** |
| *Right CA1* | **-.363** | **.049** | -.257 | .170 | -.136 | .474 | -.321 | .084^ |
| *Left CA3* | -.258 | .168 | -.180 | .340 | -.114 | .548 | -.204 | .279 |
| *Right CA3* | -.268 | .153 | -.285 | .127 | -.101 | .596 | -.237 | .207 |
| *Left CA4* | -.313 | .092^ | -.284 | .129 | -.119 | .532 | -.309 | .097^ |
| *Right CA4* | -.241 | .208 | -.174 | .365 | .075 | .700 | -.187 | .330 |
| *Left GC-ML-DG* | -.303 | .103 | -.260 | .165 | -.074 | .699 | -.283 | .130 |
| *Right GC-ML-DG* | -.209 | .276 | -.159 | .410 | .097 | .617 | -.155 | .421 |
| *Left subiculum* | -.326 | .079^ | -.073 | .702 | -.121 | .525 | -.226 | .230 |
| *Right subiculum* | -.226 | .230 | -.032 | .866 | .029 | .880 | -.125 | .510 |
| *Left presubiculum* | -.034 | .860 | .098 | .614 | .169 | .381 | .077 | .693 |
| *Right presubiculum* | -.228 | .226 | -.075 | .693 | -.079 | .680 | -.155 | .414 |
| *Left parasubiculum* | -.310 | .095^ | -.187 | .322 | -.142 | .454 | -.262 | .162 |
| *Right parasubiculum* | -.208 | .271 | -.025 | .895 | -.003 | .986 | -.081 | .670 |
| *Left HATA* | -.081 | .669 | .110 | .561 | .047 | .806 | .011 | .956 |
| *Right HATA* | -.118 | .536 | .024 | .901 | .021 | .914 | -.012 | .950 |
| *Left fimbria* | .181 | .338 | .193 | .307 | .272 | .147 | .232 | .216 |
| *Right fimbria* | .080 | .676 | .226 | .230 | .273 | .145 | .191 | .312 |
| *Note*: DES = Dissociative Experiences Scale; DP/DR = Depersonalisation/derealisation subscale of the Dissociative Experiences Scale; CA = cornu ammonis; GC-ML-DG = granule cell molecular layer of the dentate gyrus; HATA = hippocampal-amygdaloid transition area; Sig. = significant p-value; ^ = 0.05<p≤0.1 | | | | | | | | |

| **Table S4*.*** *Correlations of hippocampal volumes with severity of dissociative symptoms within DID while controlling for comorbidity.* | | | | | | | | | |
| --- | --- | --- | --- | --- | --- | --- | --- | --- | --- |
|  |  | **Amnesia** | | **Absorption** | | **DP/DR** | | **DES total** | |
|  |  | **r** | **Sig** | **r** | **Sig** | **r** | **Sig** | **r** | **Sig** |
| **Left hippocampus volume** | | | | | | | | | |
| **Controlling for:** | |  |  |  |  |  |  |  |  |
|  | All comorbidity^1^ | -.204 | .299 | -.062 | .753 | .004 | .985 | -.128 | .516 |
|  | DID NOS | -.179 | .362 | -.027 | .891 | .064 | .746 | -.084 | .671 |
|  | Somatoform disorder | -.202 | .302 | -.096 | .625 | .025 | .900 | -.140 | .476 |
|  | Major depression | -.175 | .372 | -.066 | .739 | -.024 | .902 | -.122 | .537 |
|  | Dysthymic disorder | -.209 | .286 | -.017 | .932 | .004 | .982 | -.109 | .582 |
|  | Specific phobias | -.231 | .237 | -.116 | .557 | .020 | .919 | -.157 | .426 |
|  | Panic disorder | -.165 | .402 | -.050 | .800 | .040 | .839 | -.094 | .634 |
|  | Anxiety disorder | -.159 | .420 | -.013 | .949 | .063 | .749 | -.073 | .711 |
|  | OCD | -.159 | .420 | -.013 | .949 | .063 | .749 | -.073 | .711 |
|  | PD NOS | -.206 | .292 | -.029 | .885 | .062 | .754 | -.102 | .605 |
|  | Mixed PD | -.204 | .297 | -.083 | .676 | .047 | ,811 | -.124 | .529 |
|  | Personality trait | -.204 | .297 | -.024 | .904 | .016 | .937 | -.099 | .615 |
|  | Eating disorder | -.192 | .328 | -.038 | .846 | .033 | .866 | -.104 | .599 |
|  | Sleeping disorder | -.212 | .279 | -.046 | .817 | .004 | .984 | -.125 | .525 |
|  | Catalepsy | -.283 | .144 | -.144 | .563 | -.043 | .829 | -.199 | .310 |
|  | Psych. Drop seizures | -.159 | .420 | -.013 | .949 | .063 | .749 | -.073 | .711 |
|  | ADD | -.177 | .368 | -.052 | .793 | .024 | .903 | -.103 | .604 |
|  |  |  |  |  |  |  |  |  |  |
| **Right hippocampus volume** | |  |  |  |  |  |  |  |  |
| **Controlling for:** | |  |  |  |  |  |  |  |  |
|  | All comorbidity^1^ | -.359 | .056^ | -.204 | .288 | -.128 | .508 | -.285 | .134 |
|  | DID NOS | -.332 | .079^ | -.170 | .377 | -.078 | .687 | -.246 | .198 |
|  | Somatoform disorder | -.342 | .070^ | -.194 | .312 | -.099 | .609 | -.267 | .161 |
|  | Major depression | -.318 | .092^ | -.206 | .284 | -.165 | .394 | -.271 | .155 |
|  | Dysthymic disorder | -.342 | .069^ | -.188 | .329 | -.100 | .605 | -.260 | .174 |
|  | Specific phobias | **-.380** | **.042** | .-253 | .186 | -.109 | .573 | -.307 | .106 |
|  | Panic disorder | -.301 | .113 | -.181 | .347 | -.072 | .711 | -.231 | .229 |
|  | Anxiety disorder | -.279 | .142 | -.123 | .525 | -.035 | .858 | -.194 | .313 |
|  | OCD | -.279 | .142 | -.123 | .525 | -.035 | .858 | -.194 | .313 |
|  | PD NOS | -.350 | .062^ | -.171 | .375 | -.078 | .686 | -.255 | .183 |
|  | Mixed PD | -.351 | .062^ | -.212 | .270 | -.088 | .648 | -.273 | .152 |
|  | Personality trait | -.345 | .067^ | -.167 | .387 | -.105 | .588 | -.251 | .189 |
|  | Eating disorder | -.335 | .075^ | -.166 | .388 | -.084 | .664 | -.245 | .200 |
|  | Sleeping disorder | **-.371** | **.048** | -.181 | .348 | -.140 | .470 | -.283 | .137 |
|  | Catalepsy | **-.412** | **.026** | -.237 | .215 | -.156 | .419 | -.331 | .079^ |
|  | Psych. Drop seizures | -.279 | .142 | -.123 | .525 | -.035 | .858 | -.194 | .313 |
|  | ADD | -.321 | .089^ | -.192 | .318 | -.104 | .590 | -.250 | .190 |
|  |  |  |  |  |  |  |  |  |  |
| **Left CA1 volume** | |  |  |  |  |  |  |  |  |
| **Controlling for:** | |  |  |  |  |  |  |  |  |
|  | All comorbidity^1^ | **-.407** | **.029** | -.278 | .145 | -.210 | .275 | **-.384** | **.040** |
|  | DID NOS | **-.383** | **.040** | -.245 | .199 | -.156 | .420 | -.344 | .067^ |
|  | Somatoform disorder | **-.404** | **.030** | -.299 | .115 | -.196 | .309 | **-.389** | **.037** |
|  | Major depression | **-.379** | **.043** | -.295 | .120 | -.268 | .160 | **-.388** | **.038** |
|  | Dysthymic disorder | **-.406** | **.029** | -.262 | .169 | -.206 | .284 | **-.368** | **.049** |
|  | Specific phobias | **-.435** | **.018** | -.334 | .076^ | -.205 | .285 | **-.416** | **.025** |
|  | Panic disorder | -.365 | .052^ | -.268 | .159 | -.174 | .365 | -.350 | .063^ |
|  | Anxiety disorder | -.359 | .056^ | -.226 | .239 | -.149 | .440 | -.330 | .080^ |
|  | OCD | -.359 | .056^ | -.226 | .239 | -.149 | .440 | -.330 | .080^ |
|  | PD NOS | **-.405** | **.029** | -.258 | .177 | -.180 | .349 | -.365 | .051^ |
|  | Mixed PD | **-.408** | **.028** | -.295 | .120 | -.184 | .340 | **-.383** | **.040** |
|  | Personality trait | **-.413** | **.026** | -.245 | .200 | -.209 | .278 | -.362 | .054^ |
|  | Eating disorder | **-.393** | **.035** | -.253 | .186 | -.181 | .348 | -.358 | .057^ |
|  | Sleeping disorder | **-.415** | **.025** | -.263 | .167 | -.217 | .257 | **-.382** | **.041** |
|  | Catalepsy | **-.474** | **.009** | -.325 | .085^ | -.255 | .181 | **-.448** | **.015** |
|  | Psych. Drop seizures | -.359 | .056^ | -.226 | .239 | -.149 | .440 | -.330 | .080^ |
|  | ADD | -.365 | .052^ | -.284 | .135 | -.204 | .288 | -.358 | .057^ |
|  |  |  |  |  |  |  |  |  |  |
| **Right CA1 volume** | | | | | | | | | |
| **Controlling for:** | |  |  |  |  |  |  |  |  |
|  | All comorbidity^1^ | **-.382** | **.041** | -.281 | .140 | -.166 | .390 | -.347 | .065^ |
|  | DID NOS | -.360 | .055^ | -.254 | .184 | -.128 | .507 | -.319 | .092^ |
|  | Somatoform disorder | **-.367** | **.050** | -.279 | .142 | -.138 | .476 | -.333 | .077^ |
|  | Major depression | -.344 | .068^ | -.295 | .121 | -.218 | .255 | -.343 | .069^ |
|  | Dysthymic disorder | **-.373** | **.046** | -.254 | .184 | -.150 | .437 | -.321 | .090^ |
|  | Specific phobias | **-.401** | **.031** | -.329 | .081^ | -.148 | .444 | **-.368** | **.049** |
|  | Panic disorder | -.327 | .083^ | -.263 | .168 | -.114 | .557 | -.298 | .116 |
|  | Anxiety disorder | -.300 | .114 | -.201 | .297 | -.071 | .716 | -.257 | .179 |
|  | OCD | -.300 | .114 | -.201 | .297 | -.071 | .716 | -.257 | .179 |
|  | Personality disorder NOS | **-.379** | **.043** | -.244 | .201 | -.109 | .573 | -.315 | .096^ |
|  | Mixed PD | **-.379** | **.043** | -.297 | .118 | -.126 | .516 | -.339 | .072^ |
|  | Personality trait | **-.369** | **.049** | -.244 | .202 | -.144 | .458 | -.313 | .098^ |
|  | Eating disorder | -.359 | .056^ | -.246 | .197 | -.125 | .518 | -.310 | .102 |
|  | Sleeping disorder | **-.383** | **.040** | -.257 | .179 | -.163 | .397 | -.336 | .075^ |
|  | Catalepsy | **-.413** | **.026** | -.297 | .118 | -.175 | .364 | **-.372** | **.047** |
|  | Psych. Drop seizures | -.300 | .114 | -.201 | .297 | -.071 | .716 | -.257 | .179 |
|  | ADD | -.343 | .068^ | -.270 | .156 | -.143 | .459 | -.312 | .099^ |
|  |  |  |  |  |  |  |  |  |  |
| **Left CA3 volume** | |  |  |  |  |  |  |  |  |
| **Controlling for:** | |  |  |  |  |  |  |  |  |
|  | All comorbidity^1^ | -.282 | .139 | -.210 | .275 | -.152 | .430 | -.235 | .219 |
|  | DID NOS | -.244 | .203 | -.161 | .404 | -.078 | .687 | -.176 | .361 |
|  | Somatoform disorder | -.268 | .160 | -.220 | .250 | -.119 | .537 | -.228 | .235 |
|  | Major depression | -.237 | .216 | -.196 | .307 | -.163 | .399 | -.208 | .279 |
|  | Dysthymic disorder | -.260 | .174 | -.189 | .325 | -.115 | .553 | -.204 | .288 |
|  | Specific phobias | -.277 | .146 | -.222 | .247 | -.120 | .536 | -.228 | .234 |
|  | Panic disorder | -.221 | .249 | -.177 | .359 | -.095 | .625 | -.176 | .360 |
|  | Anxiety disorder | -.185 | .338 | -.118 | .544 | -.047 | .808 | -.128 | .509 |
|  | OCD | -.185 | .338 | -.118 | .544 | -.047 | .808 | -.128 | .509 |
|  | PD NOS | -.268 | .160 | -.171 | .374 | -.098 | .614 | -.199 | .300 |
|  | Mixed PD | -.270 | .156 | -.214 | .264 | -.104 | .591 | -.218 | .256 |
|  | Personality trait | -.260 | .173 | -.173 | .370 | -.117 | .544 | -.199 | .301 |
|  | Eating disorder | -.252 | .187 | -.167 | .387 | -.102 | .599 | -.189 | .325 |
|  | Sleeping disorder | -.288 | .130 | -.180 | .351 | -.156 | .419 | -.226 | .238 |
|  | Catalepsy | -.339 | .072^ | -.247 | .197 | -.183 | .341 | -.285 | .135 |
|  | Psych. Drop seizures | -.185 | .338 | -.118 | .544 | -.047 | .808 | -.128 | .509 |
|  | ADD | -.314 | .097^ | -.171 | .374 | -.108 | .575 | -.223 | .244 |
| **Right CA3 volume** | |  |  |  |  |  |  |  |  |
| **Controlling for:** | |  |  |  |  |  |  |  |  |
|  | All comorbidity^1^ | -.290 | .127 | -.316 | .095^ | -.137 | .479 | -.268 | .160 |
|  | DID NOS | -.278 | .144 | -.298 | .117 | -.120 | .536 | -.257 | .178 |
|  | Somatoform disorder | -.263 | .168 | -.262 | .170 | -.097 | .617 | -.221 | .249 |
|  | Major depression | -.247 | .197 | -.304 | .109 | -.148 | .444 | -.242 | .206 |
|  | Dysthymic disorder | -.264 | .166 | -.309 | .103 | -.093 | .633 | -.238 | .213 |
|  | Specific phobias | -.290 | .127 | -.338 | .073^ | -.107 | .579 | -.266 | .162 |
|  | Panic disorder | -.227 | .236 | -.289 | .129 | -.079 | .686 | -.209 | .276 |
|  | Anxiety disorder | -.205 | .287 | -.237 | .215 | -.041 | .832 | -.174 | .366 |
|  | OCD | -.205 | .287 | -.237 | .215 | -.041 | .832 | -.174 | .366 |
|  | PD NOS | -.270 | .157 | -.287 | .132 | -.102 | .600 | -.237 | .215 |
|  | Mixed PD | -.275 | .148 | -.312 | .100 | -.093 | .632 | -.247 | .197 |
|  | Personality trait | -.268 | .160 | -.287 | .131 | -.101 | .603 | -.238 | .213 |
|  | Eating disorder | -.262 | .170 | -.276 | .148 | -.090 | .641 | -.225 | .240 |
|  | Sleeping disorder | -.336 | .075^ | -.296 | .119 | -.188 | .328 | -.291 | .126 |
|  | Catalepsy | -.324 | .087^ | -.335 | .076^ | -.147 | .445 | -.294 | .121 |
|  | Psych. Drop seizures | -.205 | .287 | -.237 | .215 | -.041 | .832 | -.174 | .366 |
|  | ADD | -.283 | .137 | -.285 | .135 | -.100 | .606 | -.240 | .209 |
|  |  |  |  |  |  |  |  |  |  |
| **Left CA4 volume** | |  |  |  |  |  |  |  |  |
| **Controlling for:** | |  |  |  |  |  |  |  |  |
|  | All comorbidity^1^ | -.332 | .079^ | .-308 | .104 | -.149 | .442 | -.335 | .076^ |
|  | DID NOS | -.304 | .108 | -.273 | .151 | -.097 | .616 | -.295 | .120 |
|  | Somatoform disorder | -.310 | .102 | -.272 | .153 | -.116 | .549 | -.300 | .114 |
|  | Major depression | -.294 | .122 | -.303 | .111 | -.166 | .388 | -.316 | .095^ |
|  | Dysthymic disorder | -.345 | .067^ | -.250 | .191 | -.164 | .397 | -.311 | .101 |
|  | Specific phobias | -.343 | .068^ | -.348 | .064^ | -.128 | .508 | -.348 | .064^ |
|  | Panic disorder | -.284 | .136 | -.283 | .137 | -.102 | .598 | -.289 | .128 |
|  | Anxiety disorder | -.287 | .131 | -.260 | .174 | -.087 | .654 | -.283 | .138 |
|  | OCD | -.287 | .131 | -.260 | .174 | -.087 | .654 | -.283 | .138 |
|  | PD NOS | -.317 | .094^ | -.284 | .136 | -.117 | .544 | -.308 | .104 |
|  | Mixed PD | -.331 | .079^ | -.331 | .080^ | -.107 | .580 | -.330 | .080^ |
|  | Personality trait | -.330 | .081^ | -.265 | .165 | -.135 | .484 | -.300 | .114 |
|  | Eating disorder | -.310 | .102 | -.272 | .154 | -.103 | .595 | -.295 | .121 |
|  | Sleeping disorder | -.350^ | -.063^ | -.286 | .133 | -.168 | .383 | -.338 | .073^ |
|  | Catalepsy | **-.399** | **.032** | -.357 | .057^ | -.190 | .325 | **-.398** | **.033** |
|  | Psych. Drop seizures | -.287 | .131 | -.260 | .174 | -.087 | .654 | -.283 | .138 |
|  | ADD | -.326 | .085^ | -.284 | .135 | -.119 | .540 | -.310 | .101 |
|  |  |  |  |  |  |  |  |  |  |
| **Right CA4 volume** | |  |  |  |  |  |  |  |  |
| **Controlling for:** | |  |  |  |  |  |  |  |  |
|  | All comorbidity^1^ | -.261 | .179 | -.197 | .314 | .052 | -.212 | -.212 | .278 |
|  | DID NOS | -.254 | .193 | -.189 | .336 | .055 | .780 | -.211 | .281 |
|  | Somatoform disorder | -.238 | .223 | -.143 | .468 | .078 | .692 | -.169 | .389 |
|  | Major depression | -.224 | .252 | -.200 | .309 | .023 | .908 | -.199 | .310 |
|  | Dysthymic disorder | -.256 | .189 | -.157 | .424 | .057 | .773 | -.186 | .342 |
|  | Specific phobias | -.269 | .167 | -.231 | .237 | .070 | .725 | -.222 | .257 |
|  | Panic disorder | -.211 | .280 | -.177 | .367 | .093 | .637 | -.167 | .395 |
|  | Anxiety disorder | -.175 | .374 | -.119 | .545 | .146 | .460 | -.119 | .546 |
|  | OCD | -.175 | .374 | -.119 | .545 | .146 | .460 | -.119 | .546 |
|  | PD NOS | -.232 | .235 | -.194 | .322 | .046 | .815 | -.198 | .312 |
|  | Mixed PD | -.250 | .199 | -.203 | .299 | .087 | .661 | -.199 | .311 |
|  | Personality trait | -.245 | .209 | -.167 | .397 | .070 | .725 | -.183 | .352 |
|  | Eating disorder | -.242 | .215 | -.163 | .407 | .092 | .640 | -.174 | .376 |
|  | Sleeping disorder | -.313 | .105 | -.182 | .355 | -.003 | .988 | -.242 | .214 |
|  | Catalepsy | -.330 | .087^ | -.247 | .205 | .011 | .956 | -.275 | .157 |
|  | Psych. Drop seizures | -.175 | .374 | -.119 | .545 | .146 | .460 | -.119 | .546 |
|  | ADD | -.217 | .268 | -.186 | .343 | .069 | .726 | -.177 | .368 |
|  |  |  |  |  |  |  |  |  |  |
| **Left GC-ML-DG volume** | |  |  |  |  |  |  |  |  |
| **Controlling for:** | |  |  |  |  |  |  |  |  |
|  | All comorbidity^1^ | -.319 | .092^ | -.218 | .140 | -.099 | .611 | -.305 | .108 |
|  | DID NOS | -.299 | .116 | -.255 | .183 | -.061 | .755 | -.276 | .147 |
|  | Somatoform disorder | -.301 | .113 | -.252 | .187 | -.071 | .713 | -.276 | .147 |
|  | Major depression | -.283 | .137 | -.280 | .142 | -.122 | .530 | -.289 | .128 |
|  | Dysthymic disorder | -.337 | .074^ | -.224 | .243 | -.119 | .540 | -.285 | .134 |
|  | Specific phobias | -.334 | .076^ | -.325 | .085^ | -.082 | .674 | -.323 | .088^ |
|  | Panic disorder | -.272 | .153 | -.259 | .174 | -.054 | .779 | -.261 | .171 |
|  | Anxiety disorder | -.272 | .154 | -.232 | .227 | -.036 | .852 | -.250 | .190 |
|  | OCD | -.272 | .154 | -.232 | .227 | -.036 | .852 | -.250 | .190 |
|  | PD NOS | -.305 | .108 | -.263 | .168 | -.076 | .694 | -.283 | .136 |
|  | Mixed PD | -.321 | .089^ | -.307 | .105 | -.060 | .756 | -.304 | .109 |
|  | Personality trait | -.318 | .093^ | -.241 | .209 | -.087 | .654 | -.272 | .153 |
|  | Eating disorder | -.299 | .115 | -.247 | .196 | -.057 | .769 | -.268 | .160 |
|  | Sleeping disorder | -.340 | .071^ | -.262 | .170 | -.122 | .530 | -.311 | .100 |
|  | Catalepsy | **-.385** | **.039** | -.330 | .081^ | -.139 | .471 | **-.367** | **.050** |
|  | Psych. Drop seizures | -.272 | .154 | -.232 | .227 | -.036 | .851 | -.250 | .190 |
|  | ADD | -.306 | .107 | -.264 | .167 | -.075 | .698 | -.281 | .140 |
|  |  |  |  |  |  |  |  |  |  |
| **Right GC-ML-DG volume** | |  |  |  |  |  |  |  |  |
| **Controlling for:** | |  |  |  |  |  |  |  |  |
|  | All comorbidity^1^ | -.229 | .241 | -.182 | .355 | .074 | .707 | -.179 | .361 |
|  | DID NOS | -.217 | .267 | -.168 | .394 | .087 | .659 | -.170 | .388 |
|  | Somatoform disorder | -.207 | .291 | -.141 | .475 | .099 | .616 | -.144 | .465 |
|  | Major depression | -.191 | .331 | -.184 | .348 | .045 | .819 | -.166 | .398 |
|  | Dysthymic disorder | -.226 | .248 | -.138 | .484 | .077 | .696 | -.154 | .434 |
|  | Specific phobias | -.237 | .225 | -.216 | .269 | .093 | .640 | -.189 | .334 |
|  | Panic disorder | -.181 | .357 | -.160 | .415 | .114 | .563 | -.135 | .492 |
|  | Anxiety disorder | -.143 | .468 | -.105 | .595 | .167 | .397 | -.087 | .660 |
|  | OCD | -.143 | .468 | -.105 | .595 | .167 | .397 | -.087 | .660 |
|  | PD NOS | -.204 | .297 | -.170 | .386 | .083 | .674 | -.161 | .413 |
|  | Mixed PD | -.219 | .262 | -.190 | .332 | .110 | .576 | -.167 | .395 |
|  | Personality trait | -.212 | .278 | .-152 | .440 | .093 | .639 | -.151 | .443 |
|  | Eating disorder | -.208 | .289 | -.148 | .453 | .114 | .568 | -.142 | .472 |
|  | Sleeping disorder | -.266 | .171 | -.163 | .407 | .034 | .865 | -.198 | .312 |
|  | Catalepsy | -.288 | .137 | -.224 | .251 | .040 | .838 | -.233 | .233 |
|  | Psych. Drop seizures | -.143 | .468 | -.105 | .595 | .167 | .397 | -.087 | .660 |
|  | ADD | -.186 | .343 | -.169 | .389 | .092 | .641 | -.145 | .461 |
|  |  |  |  |  |  |  |  |  |  |
| **Left subiculum volume** | |  |  |  |  |  |  |  |  |
| **Controlling for:** | |  |  |  |  |  |  |  |  |
|  | All comorbidity^1^ | -.327 | .083^ | -.074 | .702 | -.123 | .523 | -.229 | .233 |
|  | DID NOS | -.312 | .099^ | -.051 | .793 | -.085 | .662 | -.199 | .302 |
|  | Somatoform disorder | -.345 | .067^ | -.131 | .497 | -.131 | .499 | -.266 | .163 |
|  | Major depression | -.307 | .106 | -.086 | .659 | -.169 | .380 | -.231 | .229 |
|  | Dysthymic disorder | -.334 | .077^ | -.066 | .734 | -.132 | .496 | -.226 | .239 |
|  | Specific phobias | -.365 | .052^ | -.137 | .480 | -.133 | .492 | -.271 | .155 |
|  | Panic disorder | -.292 | .124 | -.064 | .741 | -.101 | .601 | -.199 | .300 |
|  | Anxiety disorder | -.312 | .100 | -.048 | .805 | -.098 | .613 | -.205 | .285 |
|  | OCD | -.312 | .100 | -.048 | .805 | -.098 | .613 | -.205 | .285 |
|  | PD NOS | -.327 | .084^ | -.075 | .701 | -.127 | .513 | -.227 | .236 |
|  | Mixed PD | -.339 | .072^ | -.104 | .593 | -.111 | .567 | -.240 | .209 |
|  | Personality trait | -.329 | .081^ | -.060 | .757 | -.126 | .515 | -.219 | .254 |
|  | Eating disorder | -.325 | .086^ | -.071 | .715 | -.199 | .539 | -.225 | .242 |
|  | Sleeping disorder | -.337 | .074^ | -.072 | .711 | -.136 | .482 | -.233 | .233 |
|  | Catalepsy | **-.380** | **.042** | -.110 | .569 | -.164 | .395 | -.278 | .144 |
|  | Psych. Drop seizures | -.312 | .100 | -.048 | .805 | -.098 | .613 | -.205 | .285 |
|  | ADD | -.295 | .120 | -.087 | .653 | -.131 | .500 | -.213 | .267 |
|  |  |  |  |  |  |  |  |  |  |
| **Right subiculum volume** | |  |  |  |  |  |  |  |  |
| **Controlling for:** | |  |  |  |  |  |  |  |  |
|  | All comorbidity^1^ | -.240 | .209 | -.049 | .800 | .006 | .975 | -.145 | .454 |
|  | DID NOS | -.214 | .265 | -.015 | .938 | .061 | .755 | -.102 | .597 |
|  | Somatoform disorder | -.230 | .229 | -.051 | .791 | .026 | .892 | -.138 | .476 |
|  | Major depression | -.193 | .317 | -.054 | .782 | -.045 | .815 | -.133 | .492 |
|  | Dysthymic disorder | -.229 | .231 | -.030 | .876 | .027 | .890 | -.125 | .518 |
|  | Specific phobias | -.251 | .189 | -.081 | .675 | .025 | .897 | -.156 | .420 |
|  | Panic disorder | -.182 | .343 | -.020 | .916 | .059 | .762 | -.090 | .643 |
|  | Anxiety disorder | -.140 | .470 | .050 | .798 | .115 | .554 | -.032 | .869 |
|  | OCD | -.140 | .470 | .050 | .798 | .115 | .554 | -.032 | .869 |
|  | PD NOS | -.229 | .231 | -.028 | .884 | .037 | .848 | -.124 | .523 |
|  | Mixed PD | -.231 | .228 | -.049 | .799 | .037 | .850 | -.132 | .496 |
|  | Personality trait | -.228 | .233 | -.018 | .926 | .024 | .900 | -.116 | .548 |
|  | Eating disorder | -.220 | .252 | -.020 | .918 | 039 | .839 | -.113 | .559 |
|  | Sleeping disorder | -.239 | .212 | -.031 | .874 | .013 | .947 | -.134 | .487 |
|  | Catalepsy | -.254 | .184 | -.050 | .795 | .011 | .956 | -.151 | .435 |
|  | Psych. Drop seizures | -.140 | .470 | .050 | .798 | .115 | .554 | -.032 | .869 |
|  | ADD | -.142 | .461 | -.064 | .741 | .013 | .947 | -.095 | .624 |
|  |  |  |  |  |  |  |  |  |  |
| **Left presubiculum volume** | |  |  |  |  |  |  |  |  |
| **Controlling for:** | |  |  |  |  |  |  |  |  |
|  | All comorbidity^1^ | -.036 | .854 | .094 | .635 | .164 | .405 | .073 | .713 |
|  | DID NOS | -.023 | .907 | .113 | .568 | .119 | .310 | .100 | .613 |
|  | Somatoform disorder | -.038 | .848 | .073 | .711 | .169 | .391 | .063 | .751 |
|  | Major depression | -.027 | .891 | .093 | .638 | .158 | .432 | .075 | .705 |
|  | Dysthymic disorder | -.060 | .761 | .162 | .409 | .133 | .500 | .085 | .669 |
|  | Specific phobias | -.045 | .820 | .074 | .709 | .167 | .395 | .063 | .752 |
|  | Panic disorder | -.013 | .947 | .098 | .619 | .178 | .364 | .091 | .646 |
|  | Anxiety disorder | -.011 | .955 | .120 | .542 | .194 | .324 | .105 | .594 |
|  | OCD | -.011 | .955 | .120 | .542 | .194 | .324 | .105 | .594 |
|  | PD NOS | -.032 | .871 | .095 | .629 | .169 | .390 | .075 | .705 |
|  | Mixed PD | -.037 | .853 | .082 | .678 | .181 | .358 | .073 | .712 |
|  | Personality trait | -.039 | .846 | .116 | .557 | .163 | .407 | .088 | .655 |
|  | Eating disorder | -.033 | .867 | .101 | .609 | .172 | .383 | .080 | .685 |
|  | Sleeping disorder | -.028 | .888 | .098 | .621 | .184 | .348 | .083 | .675 |
|  | Catalepsy | -.052 | .794 | .087 | .660 | .160 | .416 | .063 | .749 |
|  | Psych. Drop seizures | -.011 | .955 | .120 | .542 | .194 | .324 | .105 | .594 |
|  | ADD | -.028 | .886 | .096 | .626 | .168 | .392 | .080 | .685 |
|  |  |  |  |  |  |  |  |  |  |
| **Right presubiculum volume** | |  |  |  |  |  |  |  |  |
| **Controlling for:** | |  |  |  |  |  |  |  |  |
|  | All comorbidity^1^ | -.237 | .215 | -.087 | .652 | -.096 | .620 | -.168 | .383 |
|  | DID NOS | -.213 | .267 | -.055 | .776 | -.044 | .820 | -.127 | .511 |
|  | Somatoform disorder | -.239 | .212 | -.117 | .545 | -.085 | .662 | -.181 | .347 |
|  | Major depression | -.208 | .279 | -.085 | .659 | -.118 | .544 | -.156 | .419 |
|  | Dysthymic disorder | -.232 | .226 | -.073 | .705 | -.084 | .664 | -.154 | .424 |
|  | Specific phobias | -.249 | .192 | -.120 | .536 | -.085 | .662 | -.182 | .345 |
|  | Panic disorder | -.212 | .269 | -.071 | .714 | -.070 | .719 | -.142 | .462 |
|  | Anxiety disorder | -.172 | .372 | -.022 | .908 | -.027 | .890 | -.095 | .623 |
|  | OCD | -.172 | .372 | -.022 | .908 | -.027 | .890 | -.095 | .623 |
|  | PD NOS | -.233 | .225 | -.070 | .718 | -.071 | .716 | -.152 | .431 |
|  | Mixed PD | -.239 | .212 | -.106 | .584 | -.068 | .728 | -.167 | .386 |
|  | Personality trait | -.228 | .235 | -.075 | .700 | -.079 | .684 | -.155 | .423 |
|  | Eating disorder | -.223 | .245 | -.067 | .730 | -.072 | .711 | -.146 | .449 |
|  | Sleeping disorder | -.226 | .238 | -.076 | .696 | -.075 | .700 | -153 | .429 |
|  | Catalepsy | -.253 | .186 | -.092 | .634 | -.098 | .614 | -.178 | .355 |
|  | Psych. Drop seizures | -.172 | .372 | -.022 | .908 | -.027 | .890 | -.095 | .623 |
|  | ADD | -.194 | .314 | -.098 | .647 | -.087 | .653 | -.141 | .466 |
|  |  |  |  |  |  |  |  |  |  |
| **Left parasubiculum volume** | |  |  |  |  |  |  |  |  |
| **Controlling for:** | |  |  |  |  |  |  |  |  |
|  | All comorbidity^1^ | -.317 | .094^ | -.196 | .307 | -.155 | .423 | -.272 | .153 |
|  | DID NOS | -.302 | .112 | -175 | .363 | .-122 | .530 | -.247 | .196 |
|  | Somatoform disorder | -.307 | .105 | -.175 | .363 | -.140 | .469 | -.255 | .182 |
|  | Major depression | -.307 | .106 | -.190 | .324 | -.153 | .428 | -.262 | .170 |
|  | Dysthymic disorder | -.319 | .092^ | -.183 | .343 | -.155 | .422 | -.262 | .170 |
|  | Specific phobias | -.338 | .073^ | -.243 | .205 | -.151 | .433 | -,297 | .118 |
|  | Panic disorder | -.289 | .128 | -.183 | .341 | -.130 | .500 | -.246 | .198 |
|  | Anxiety disorder | -.288 | .130 | -.161 | .403 | -.114 | .555 | -.237 | .217 |
|  | OCD | -.288 | .130 | -.161 | .403 | -.114 | .555 | -.237 | .217 |
|  | PD NOS | -.311 | .100 | -.190 | .324 | -.148 | .443 | -.263 | .168 |
|  | Mixed PD | -.322 | .088^ | -.220 | .252 | -.133 | .492 | -.276 | .147 |
|  | Personality trait | -.311 | .101 | -.183 | .341 | -.144 | .457 | -.260 | .174 |
|  | Eating disorder | -.305 | .108 | -.174 | .367 | -.131 | .500 | -.249 | .193 |
|  | Sleeping disorder | -.340 | .071^ | -.187 | .332 | -.183 | .341 | -.284 | .135 |
|  | Catalepsy | **-.370** | **.048** | -.234 | .221 | -.192 | .318 | -.323 | ,088^ |
|  | Psych. Drop seizures | -.288 | .130 | -.161 | .403 | -.114 | .555 | -.237 | .217 |
|  | ADD | -.347 | .065^ | -.182 | .344 | -.139 | .473 | -.273 | .152 |
|  |  |  |  |  |  |  |  |  |  |
| **Right parasubiculum volume** | |  |  |  |  |  |  |  |  |
| **Controlling for:** | |  |  |  |  |  |  |  |  |
|  | All comorbidity^1^ | -.260 | .174 | -.076 | .697 | -.072 | .711 | -.139 | .473 |
|  | DID NOS | -.180 | .349 | .018 | .926 | .074 | .702 | -.019 | .921 |
|  | Somatoform disorder | -.209 | .276 | -.032 | .869 | -.004 | .983 | -.086 | .658 |
|  | Major depression | -.179 | .352 | -.040 | .838 | -.059 | .762 | -.082 | .672 |
|  | Dysthymic disorder | -.220 | .252 | -.007 | .973 | -.020 | .919 | -.080 | .679 |
|  | Specific phobias | -.218 | .256 | -.049 | .801 | -.006 | .976 | -.095 | .625 |
|  | Panic disorder | -.175 | .363 | -.017 | .932 | .016 | .934 | -.054 | .781 |
|  | Anxiety disorder | -.104 | .592 | .072 | .712 | .094 | .626 | .034 | .861 |
|  | OCD | -.104 | .592 | .072 | .712 | .094 | .626 | .034 | .861 |
|  | PD NOS | -.208 | .280 | -.027 | .889 | -.007 | .973 | -.082 | .671 |
|  | Mixed PD | -.212 | .270 | -.040 | .836 | .003 | .987 | -.086 | .656 |
|  | Personality trait | -.214 | .264 | -.001 | .997 | -.011 | .953 | -.066 | .735 |
|  | Eating disorder | -.200 | .298 | .005 | .978 | .023 | .905 | -.051 | .794 |
|  | Sleeping disorder | -.226 | .239 | -.023 | .904 | -.028 | .887 | -.094 | .629 |
|  | Catalepsy | -.277 | .146 | -.077 | .693 | -.059 | .761 | -.146 | .451 |
|  | Psych. Drop seizures | -.104 | .592 | .072 | .712 | .094 | .626 | .034 | .861 |
|  | ADD | -.186 | .333 | -.034 | .862 | -.009 | .965 | -.071 | .715 |
|  |  |  |  |  |  |  |  |  |  |
| **Left HATA volume** | |  |  |  |  |  |  |  |  |
| **Controlling for:** | |  |  |  |  |  |  |  |  |
|  | All comorbidity^1^ | -.106 | .584 | .084 | .664 | .005 | .978 | -.021 | .913 |
|  | DID NOS | -.077 | .690 | .117 | .545 | .058 | .765 | .019 | .921 |
|  | Somatoform disorder | -.080 | .680 | .122 | .527 | .048 | .804 | .016 | .936 |
|  | Major depression | -.039 | .843 | .103 | .596 | -.016 | .934 | .015 | .937 |
|  | Dysthymic disorder | -.071 | .713 | .093 | .633 | .067 | .732 | .009 | .961 |
|  | Specific phobias | -.091 | .641 | .090 | .642 | .045 | .816 | -.002 | .993 |
|  | Panic disorder | -.006 | .976 | .143 | .458 | .094 | .629 | .075 | .699 |
|  | Anxiety disorder | -.026 | .894 | .163 | .398 | .096 | .621 | .071 | .716 |
|  | OCD | -.026 | .894 | .163 | .398 | .096 | .621 | .071 | .716 |
|  | PD NOS | -.093 | .632 | .129 | .506 | .079 | .684 | .019 | .920 |
|  | Mixed PD | -.083 | .668 | .104 | .592 | .051 | .794 | .008 | .966 |
|  | Personality trait | -.083 | .669 | .127 | .513 | .043 | .826 | .021 | .913 |
|  | Eating disorder | -.081 | .675 | .111 | .566 | .047 | .808 | .011 | .955 |
|  | Sleeping disorder | -.073 | .707 | .109 | .572 | .064 | .743 | .018 | .926 |
|  | Catalepsy | -.046 | .812 | .149 | .441 | .087 | .654 | .052 | .790 |
|  | Psych. Drop seizures | -.026 | .894 | .163 | .398 | .096 | .621 | .071 | .716 |
|  | ADD | -.029 | .882 | .098 | .615 | .038 | .843 | .032 | .869 |
|  |  |  |  |  |  |  |  |  |  |
| **Right HATA volume** | |  |  |  |  |  |  |  |  |
| **Controlling for:** | |  |  |  |  |  |  |  |  |
|  | All comorbidity^1^ | -.183 | .342 | -.039 | .841 | -.070 | .717 | -.083 | .668 |
|  | DID NOS | -.124 | .522 | .018 | .927 | .011 | .957 | -.022 | .911 |
|  | Somatoform disorder | -.115 | .553 | .039 | .842 | .023 | .907 | -.004 | .984 |
|  | Major depression | -.068 | .726 | .006 | .977 | -.065 | .736 | -.008 | .966 |
|  | Dysthymic disorder | -.102 | .597 | -.010 | .960 | .050 | .797 | -.014 | .942 |
|  | Specific phobias | -.135 | .487 | -.014 | .942 | .017 | .929 | -.033 | .865 |
|  | Panic disorder | -.058 | .766 | .042 | .829 | .056 | .773 | .039 | .841 |
|  | Anxiety disorder | -.059 | .761 | .077 | .692 | .073 | .707 | .052 | .787 |
|  | OCD | -.059 | .761 | .077 | .692 | .073 | .707 | .052 | .787 |
|  | PD NOS | -.115 | .551 | .020 | .920 | .013 | .946 | -.014 | .941 |
|  | Mixed PD | -.122 | .527 | .006 | .974 | .029 | .882 | -.018 | .928 |
|  | Personality trait | -.117 | .544 | .021 | .915 | .022 | .911 | -.014 | .941 |
|  | Eating disorder | -.120 | .536 | .021 | .913 | .018 | .924 | -.015 | .938 |
|  | Sleeping disorder | -.126 | .516 | .025 | .899 | .010 | .957 | -.017 | .929 |
|  | Catalepsy | -.092 | .636 | .051 | .792 | .051 | .793 | .019 | .921 |
|  | Psych. Drop seizures | -.059 | .761 | .077 | .692 | .073 | .707 | .052 | .787 |
|  | ADD | -.067 | .730 | .009 | .964 | .012 | .953 | .009 | .963 |
|  |  |  |  |  |  |  |  |  |  |
| **Left fimbria volume** | |  |  |  |  |  |  |  |  |
| **Controlling for:** | |  |  |  |  |  |  |  |  |
|  | All comorbidity^1^ | .183 | .342 | .196 | .308 | .278 | .144 | .236 | .217 |
|  | DID NOS | .171 | .375 | .182 | .346 | .257 | .179 | .217 | .257 |
|  | Somatoform disorder | .178 | .355 | .183 | .342 | .270 | .157 | .226 | .239 |
|  | Major depression | .196 | .309 | .190 | .324 | .260 | .173 | .234 | .221 |
|  | Dysthymic disorder | .153 | .427 | .278 | .144 | .229 | .233 | .244 | .201 |
|  | Specific phobias | .180 | .351 | .190 | .323 | .271 | .155 | .231 | .229 |
|  | Panic disorder | .167 | .388 | .190 | .324 | .265 | .164 | .222 | .246 |
|  | Anxiety disorder | .212 | .270 | .215 | .264 | .296 | .119 | .264 | .166 |
|  | OCD | .212 | .270 | .215 | .264 | .296 | .119 | .264 | .166 |
|  | PD NOS | .202 | .292 | .171 | .375 | .236 | .218 | .222 | .246 |
|  | Mixed PD | .182 | .345 | .198 | .302 | .271 | .156 | .234 | .222 |
|  | Personality trait | .181 | .347 | .203 | .290 | .270 | .157 | .240 | .210 |
|  | Eating disorder | .201 | .297 | .219 | .253 | .297 | .118 | .263 | .168 |
|  | Sleeping disorder | .203 | .290 | .192 | .318 | .311 | .100 | .250 | .190 |
|  | Catalepsy | .159 | .410 | .173 | .368 | .254 | .185 | .212 | .270 |
|  | Psych. Drop seizures | .212 | .270 | .215 | .264 | .296 | .119 | .264 | .166 |
|  | ADD | .257 | .179 | .181 | .347 | .268 | .159 | .263 | .168 |
|  |  |  |  |  |  |  |  |  |  |
| **Right fimbria volume** | |  |  |  |  |  |  |  |  |
| **Controlling for:** | |  |  |  |  |  |  |  |  |
|  | All comorbidity^1^ | .076 | .694 | .222 | .247 | .269 | .158 | .187 | .331 |
|  | DID NOS | .088 | .650 | .237 | .215 | .297 | .118 | .209 | .277 |
|  | Somatoform disorder | .085 | .660 | .259 | .175 | .277 | .145 | .209 | .277 |
|  | Major depression | .086 | .658 | .224 | .242 | .269 | .158 | .192 | .319 |
|  | Dysthymic disorder | .090 | .642 | .215 | .263 | .296 | .118 | .191 | .322 |
|  | Specific phobias | .087 | .652 | .250 | .190 | .277 | .146 | .204 | .289 |
|  | Panic disorder | .079 | .682 | .226 | .239 | .273 | .152 | .192 | .319 |
|  | Anxiety disorder | .157 | .415 | .293 | .123 | .341 | .070^ | .274 | .150 |
|  | OCD | .157 | .415 | .293 | .123 | .341 | .070^ | .274 | .150 |
|  | PD NOS | .101 | .601 | .202 | .292 | .232 | .226 | .179 | .352 |
|  | Mixed PD | .085 | .662 | .252 | .189 | .267 | .161 | .200 | .298 |
|  | Personality trait | .079 | .682 | .230 | .229 | .272 | .153 | .194 | .313 |
|  | Eating disorder | .098 | .612 | .258 | .177 | .302 | .111 | .225 | .241 |
|  | Sleeping disorder | .087 | .654 | .225 | .240 | .290 | .127 | .197 | .305 |
|  | Catalepsy | .077 | .692 | .227 | .237 | .276 | .146 | .192 | .318 |
|  | Psych. Drop seizures | .157 | .415 | .293 | .123 | .341 | .070^ | .274 | .150 |
|  | ADD | .146 | .449 | .215 | .262 | .270 | .157 | .219 | .253 |
|  |  |  |  |  |  |  |  |  |  |
| *Note*: DP/DR = depersonalisation/derealisation; DID NOS = dissociative identity disorder not otherwise specified; OCD = obsessive-compulsive disorder; PD NOS = personality disorder not otherwise specified; mixed PD = mixed personality disorder; ADD = attention deficit disorder; psych. drop seizures = psychogenic drop-seizures; GC-ML-DG = granule cell molecular layer of the dentate gyrus; HATA = hippocampal-amygdaloid transition area; ^1^ = excluding post-traumatic stress disorder. | | | | | | | | | |

| **Table S5*.*** *Correlations of hippocampal volumes with severity of total childhood trauma and composite scores for abuse category within DID.* | | | | | | | | | | | | |
| --- | --- | --- | --- | --- | --- | --- | --- | --- | --- | --- | --- | --- |
|  | **TEC total** | | **Emotional abuse** | | **Emotional neglect** | | **Physical abuse** | | **Sexual abuse** | | **Sexual harassment** | |
|  | ***r*** | **Sig.** | ***r*** | **Sig.** | ***r*** | **Sig.** | ***r*** | **Sig.** | ***r*** | **Sig.** | ***r*** | **Sig.** |
| **Hippocampal volumes** | | | | | | | | | | | | |
| *Left hippocampus* | .207 | .300 | -.082 | .686 | **-.442** | **.021** | .345 | .078^ | .144 | .473 | .049 | .812 |
| *Right hippocampus* | .306 | .121 | .054 | .787 | **-.431** | **.025** | .289 | .143 | .160 | .426 | -.166 | .419 |
| *Left CA1* | .336 | .086^ | -.009 | .966 | **-.408** | **.035** | .353 | .071^ | .254 | .200 | -.013 | .951 |
| *Right CA1* | .287 | .147 | .053 | .791 | **-.392** | **.043** | .308 | .118 | .124 | .537 | -.152 | .458 |
| *Left CA3* | .018 | .929 | .077 | .704 | -.280 | .158 | .314 | .110 | .018 | .928 | .072 | .726 |
| *Right CA3* | .218 | .275 | .199 | .320 | **-.411** | **.033** | .137 | .497 | .038 | .850 | -.259 | .202 |
| *Left CA4* | .213 | .286 | -.053 | .794 | **-.446** | **.020** | .312 | .113 | .068 | .737 | .277 | .170 |
| *Right CA4* | **.402** | **.042** | .175 | .393 | **-.462** | **.017** | .241 | .236 | .119 | .561 | -.070 | .739 |
| *Left GC-ML-DG* | .274 | .166 | -.053 | .793 | **-.460** | **.016** | .331 | .092^ | .122 | .543 | .255 | .208 |
| *Right GC-ML-DG* | **.455** | **.019** | .142 | .489 | **-.469** | **.016** | .233 | .251 | .107 | .602 | -.083 | .693 |
| *Left subiculum* | .200 | .318 | -.193 | .336 | -.345 | .078^ | .210 | .294 | .228 | .252 | -.045 | .826 |
| *Right subiculum* | .160 | .425 | .015 | .940 | -.309 | .117 | .124 | .538 | .053 | .794 | -.197 | .334 |
| *Left presubiculum* | .047 | .816 | -.253 | .203 | -.333 | .090^ | .084 | .679 | .124 | .536 | .023 | .912 |
| *Right presubiculum* | .070 | .729 | -.259 | .193 | -.271 | .171 | -.023 | .909 | -.086 | .669 | -.002 | .991 |
| *Left parasubiculum* | .309 | .116 | -.112 | .578 | -.122 | .545 | .307 | .119 | .268 | .177 | .266 | .190 |
| *Right parasubiculum* | -.014 | .946 | -.122 | .544 | .048 | .812 | .107 | .594 | .219 | .273 | .107 | .604 |
| *Left HATA* | -.043 | .833 | -.209 | .296 | -.232 | .244 | -.076 | .705 | -.016 | .937 | -.065 | .752 |
| *Right HATA* | -.017 | .934 | -.063 | .757 | -.072 | .723 | -.132 | .510 | -.132 | .511 | .036 | .860 |
| *Left fimbria* | .268 | .177 | .106 | .597 | -.073 | .717 | .005 | .980 | .148 | .462 | .194 | .344 |
| *Right fimbria* | -.027 | .893 | .074 | .714 | .041 | .838 | -.035 | .861 | -.167 | .405 | **.397** | **.045** |
| *Note*: TEC = Traumatic Experiences Checklist; CA = cornu ammonis; GC-ML-DG = granule cell molecular layer of the dentate gyrus; HATA = hippocampal-amygdaloid transition area; Sig. = significant p-value; ^ = 0.05<p≤0.1 | | | | | | | | | | | | |


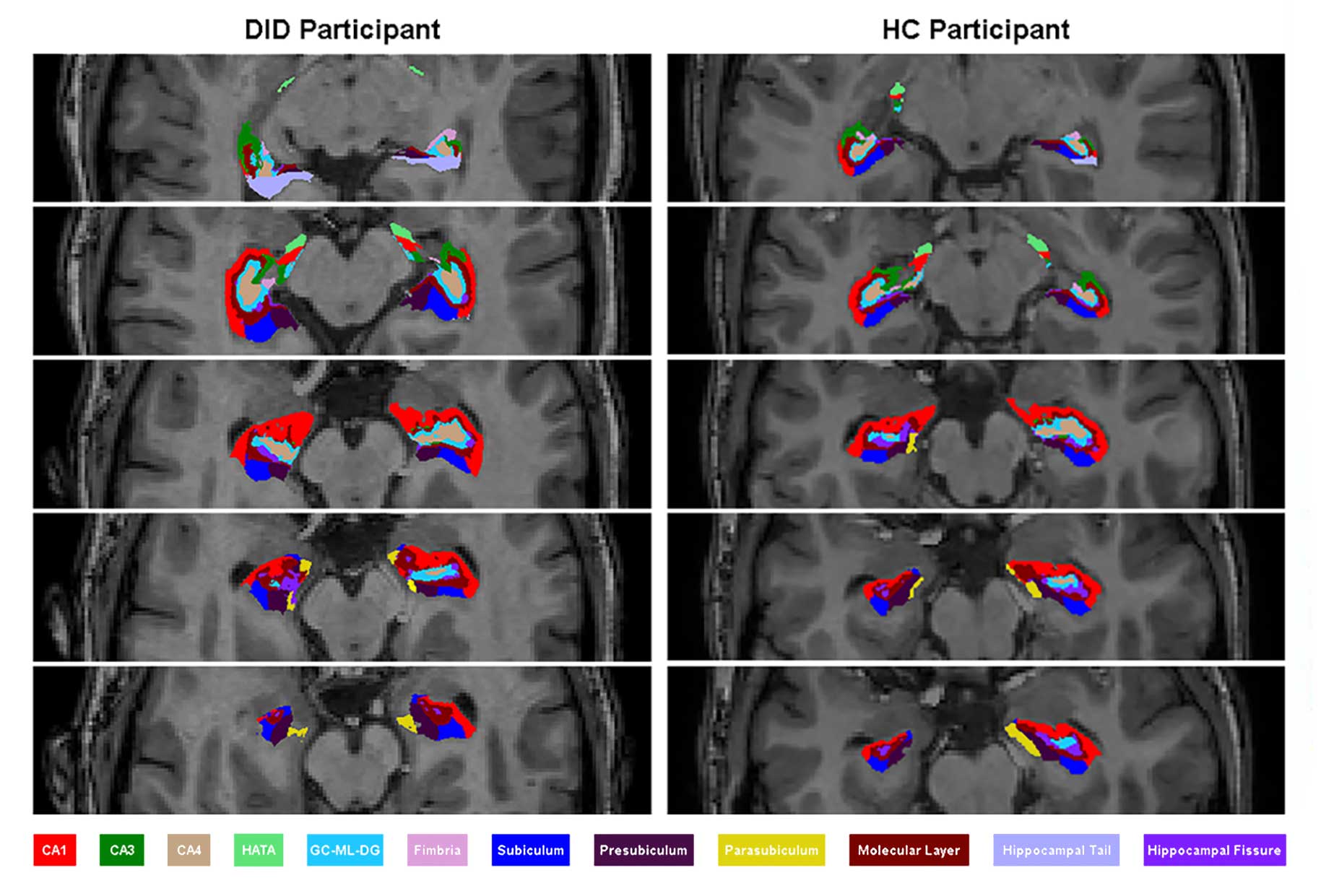


**Figure S1.** Axial slices showcasing the hippocampus from a DID participant and a HC participant.
